# Fragility Fractures of the Acetabulum: Current Concepts for Improving Patients’ Outcomes

**DOI:** 10.1007/s43465-022-00653-0

**Published:** 2022-05-26

**Authors:** Giuseppe Toro, Adriano Braile, Annalisa De Cicco, Raffaele Pezzella, Francesco Ascione, Antonio Benedetto Cecere, Alfredo Schiavone Panni

**Affiliations:** 1 Department of Medical and Surgical Specialties and Dentistry, University of Campania “Luigi Vanvitelli”, 80138 Naples, Italy; 2 Department of Clinical Sciences and Translational Medicine, University of Rome Tor Vergata, 00133 Rome, Italy; 3 Unit of Orthopedics and Traumatology, AORN San Giuseppe Moscati, 83100 Avellino, Italy; 4 Department of Orthopaedic and Traumatology Surgery, Ospedale Buon Consiglio Fatebenefratelli, 80123 Naples, Italy

**Keywords:** Acetabular fracture, Elderly, Fragility fracture, Open reduction and internal fixation, Total hip arthroplasty, Percutaneous fixation, Osteoporosis, Hip fracture, Mortality, Plate

## Abstract

The incidence of fragility fractures of the acetabulum (FFA) is constantly increasing. Generally, these fractures are related to a fall on the greater trochanter involving the anterior column. The management of FFA is extremely difficult considering both patients’ comorbidities and poor bone quality. Both non-operative and several operative treatment protocols are available, and the choice among them is still ambiguous. The proposed surgical techniques for FFA [namely open reduction and internal fixation (ORIF), percutaneous fixation and total hip arthroplasty (THA)] are associated with a high complication rate. The treatment with the higher early mortality is the ORIF + THA, while the one with the lowest is the non-operative. However, at longer follow-up, this difference dreadfully change is becoming the opposite. Frequently ORIF, percutaneous fixation, and non-operative treatment need a subsequent re-operation through a THA. This latter could be extremely difficult, because of poor bone quality, acetabular mal union/non-union, bone gaps and hardware retention. However, the outcomes of each of the proposed treatment are mostly poor and controverted; therefore, a comprehensive patient evaluation and an accurate fracture description are required to appropriately manage acetabular fracture in the elderly.

## Introduction

The constant increase in life expectancy led to a growing incidence of fragility fractures [1–4]. Recently, a constant increase of fragility fractures of the acetabulum (FFA) has been observed [5]. Particularly, a 2.4-fold increase in the incidence of acetabular fractures in patients over 60 years of age during the last 3 decades was observed, making this population one of the most commonly affected (about 24% of all acetabular fractures) [6]. As a definition, FFA are due to a fall from a standing height, with a subsequent impact on the greater trochanter. The resulting anteromedial force commonly leads to a fracture of the anterior column and/or the quadrilateral plate with a medialization of the femoral head and a supero-medial roof impaction [6]. The management of FAA is still a matter of debate. Particularly, indications for non-operative versus operative treatment, the reliability of surgical fixation in an osteoporotic bone and the safety of complex joint reconstructive procedures (i.e., revision arthroplasties) are some of the unmet needs. Moreover, regardless of the treatment choice, final outcomes are mostly poor both in terms of function and mortality (Table 1). The aim of the present study is to aid the orthopaedic surgeon in the treatment decision making for fragility acetabular fractures through a comprehensive literature review, focusing on the technical tips that may aid to improve patient’s outcomes.

**Table 1:** Treatment-related mortality risk. Adapted from Daurka et al.^9^

Procedure	Mortality rate (%)	Mortality follow-up (months)
Conservative	12	52
ORIF	15.3	42.2
ORIF + THA	13.15	33.3
Percutaneous Fixation	30.5	121.8

*ORIF* open reduction and internal fixation, *THA* total hip arthroplasty

## Patient Evaluation and Fracture Pattern

**Fig. 1:**
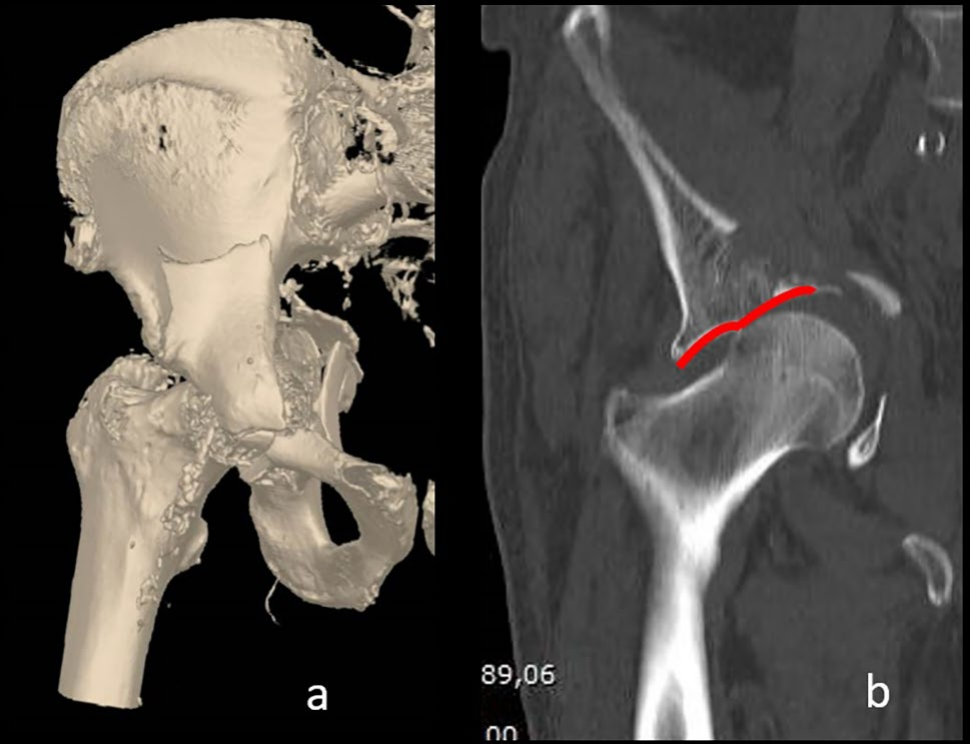
**a** A 3D reconstruction of a two columns fracture with the involvement of the quadrilateral plate occurred in a 75-year-old male. **b** Coronal reconstruction of a CT scan. Please note in red the “gull sing” that represent the result of the supero-medial impaction of the femoral head

FFA presents some differences from acetabular fractures observed in the young. In fact, in the elderly, most of acetabular fractures are related to a lateral compression force on the greater trochanter transmitted antero-medially to the anterior column, the anterior wall and the quadrilateral plate (Fig. 1) [6–8]. This characteristic mechanism of fracture explains the high incidence anterior column fractures both elementary and associated [4]. Furthermore, because of poor bone quality, the FFA is associated to an increased incidence of both femoral head injury and posterior hip dislocation related to a more severe posterior wall involvement (i.e. marginal impaction or comminution) [5, 9, 10]. These observations underline the troublesome need of both appropriately diagnose and treat fragility acetabular fractures. As a rule, a comprehensive evaluation of an elderly patient after a fall from a standing height is mandatory, investigating on both the femoral neck and the acetabulum, and to adequately manage the patient an appropriate evaluation of pre-fracture patients’ walking ability is recommendable.

The clinics of a patient with an acetabular fracture depend on the fracture displacement, varying from the absence of pain and normal range of motion (ROM) to intractable pain, lower limb discrepancy and restricted ROM.

In case of a suspected acetabular fracture, a standardized radiograph protocol, based on an anteroposterior (AP) and Judet oblique views (both obturator and iliac), must be obtained [11]. These X-rays are generally able to identify and classify the fracture. However, CT scan is useful to improve fracture diagnosis and classification. In fact, some characteristics of the fractures (i.e., articular incongruities, nondisplaced fractures, intra-articular fragments, femoral head subluxation or quadrilateral plate impaction) are easily observed using a CT scan. Moreover, CT with 3D reconstructions can help to visualize complex fractures and to plan the surgical procedure, being able to enhance diagnostic and therapeutic accuracy [12]. However, considering the mechanism of fracture and the poor bone quality, fragility acetabular fractures might be nondisplaced and difficult to diagnose. Therefore, in case of high suspicious of FFA with inconclusive X-ray and CT scans, an MRI or a bone scan should be used to identify the fracture [11, 13, 14].

## The Management of FFA

In the elderly, the appropriate management of an acetabular fracture should consider not only the fracture pattern and the available treatment options, but also the patient’s health status and pre-fracture mobility. Therefore, a comprehensive patient’s history must be collected. Different treatment options are available both non-operative and operative. However, recently, a shift in paradigm from the conservative to the operative treatment could be observed, with the final purpose of improving patient’s early mobility and lowering bed rest complications.

### Non-operative Treatment

The non-operative treatment of elderly patients with FFA might be associated to poor outcomes, in terms of both bed rest complications and joint function. Therefore, it should be indicated only in selected fractures patterns and patients [15–17]. Patients with severe comorbidities or with a severely impaired pre-injury mobility are those most eligible for conservative management. Moreover, also minimally displaced fractures (including anterior column and transverse ones) could be managed non-operatively, thanks to the intrinsic fracture stability [17–19]. According Lovric et al., functional outcomes and bed rest were lower in conservatively treated transverse fractures than anterior column/wall ones [20]. The relevance of fracture pattern in patient’s outcomes was further underlined by Heeg et al. in a study on 57 conservatively treated FFA [21]. In fact, the authors observed that patients with acetabular roof impaction and a displacement over 2 mm reported the worst outcomes [21]. However, fracture displacement should not be considered a mandatory factor for surgical indication. In fact, fractures with a secondary congruence of the hip joint (defined as congruence of the femoral head with the acetabular roof on antero-posterior and Judet views without traction [22]) might be considered for non-operative treatment. The secondary congruence could be commonly observed in both column fractures, where a congruency between the femoral head and the acetabulum might be observed despite the lack of a continuity of the articular surface with the hemipelvis [23].

Skeletal traction should be avoided, considering the high the high risk of pin-related complications (i.e. pin site infections or pin pull-out), the unviable reduction due to the rotational deforming forces that acts on the acetabular fractures, the need of prolonged bed immobilization and poor bone quality [18, 24, 25]. Therefore, also those patients non-operatively treated should be early mobilized, while partial weight-bearing initiated as soon as possible depending on patient’ tolerance. However, a constant evaluation of the fracture stability through several radiographs is suggested during the entire follow-up period to early identify any secondary displacement that may require a change in the management [5, 14, 17].

### Operative Treatment

#### Osteosynthesis

##### Open Reduction and Internal Fixation (ORIF)

The FFA are associated frequently associated to bad outcomes because of some of the conditions associated to the aging, like osteoporosis and low level of activity, are considered risk factors for poor results [26]. However, although the clinical outcomes are generally worse than those reported in the younger population, ORIF represents the gold standard of treatment for most displaced FFA [18]. Anyway, as a rule, considering patient’s comorbidities, fracture comminution, and poor bone quality, also a non-anatomic reduction can ensure optimal results in terms of both fracture healing and early mobilization, that represent mandatory goals in the elderly [27–29].

Obviously, the surgical plan of an ORIF of an FFA should start from the surgical approach. The surgical approaches available for the treatment of fragility acetabular fractures are the same described for those occurring after a high-energy trauma [30]. However, considering the comorbidities and the singular fracture pattern observable in the elderly, whenever possible non-extensile approaches should be preferred over combined and extensile ones, because of these latter are associated with longer surgical times and higher complications rate [31]. In fractures involving the anterior wall, the anterior column, or the quadrilateral plate, an ilioinguinal approach is generally preferred. However, the anterior column and the quadrilateral plate fractures might be treated using the Stoppa approach. On the other hand, the Kocher–Langenbeck approach is recommended for posterior column or posterior wall fractures [31].

**Fig. 2:**
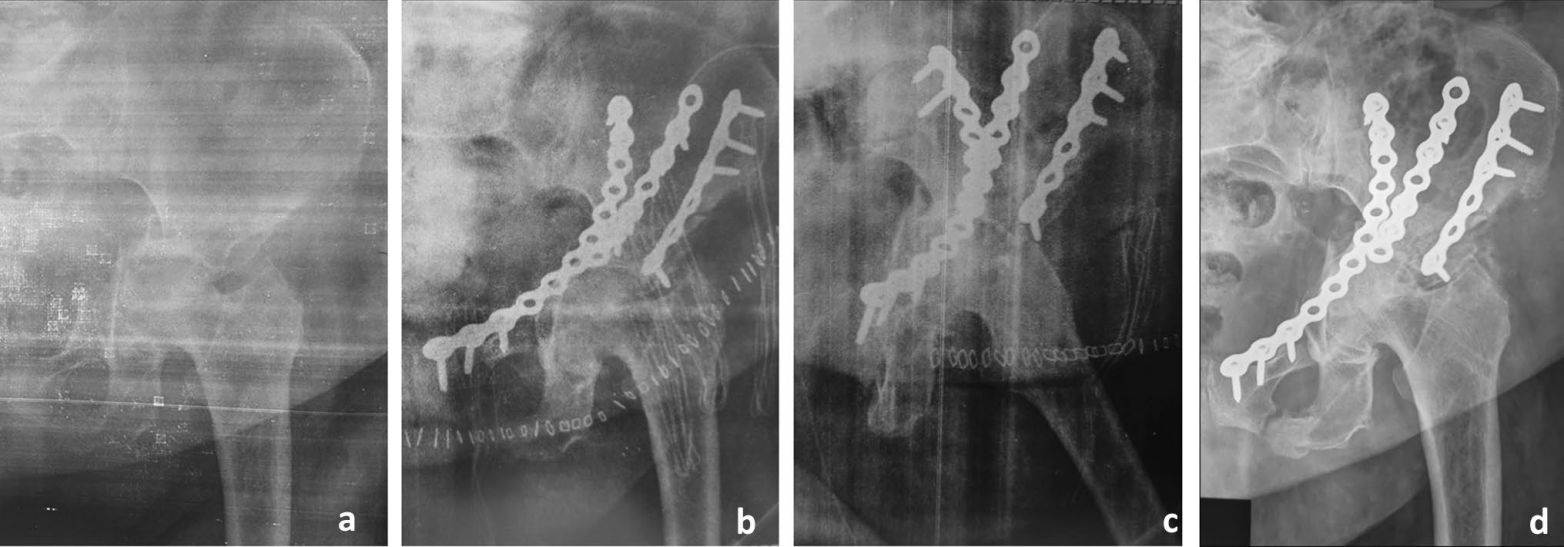
**a** Anteroposterior X-ray view of a fragility both column fracture occurred in an 84-year-old female. Please note the degree of fracture fragmentation and the involvement of the quadrilateral plate. **b** and **c** Anteroposterior and oblique postoperative X-rays. Because of the patient started to complain respiratory failure during the procedure, a non-anatomic reduction of the quadrilateral plate was accepted. **d** Anteroposterior X-ray at 1 month of the surgery showing reduction loosening of the quadrilateral plate and femoral head medialization

**Fig. 3:**
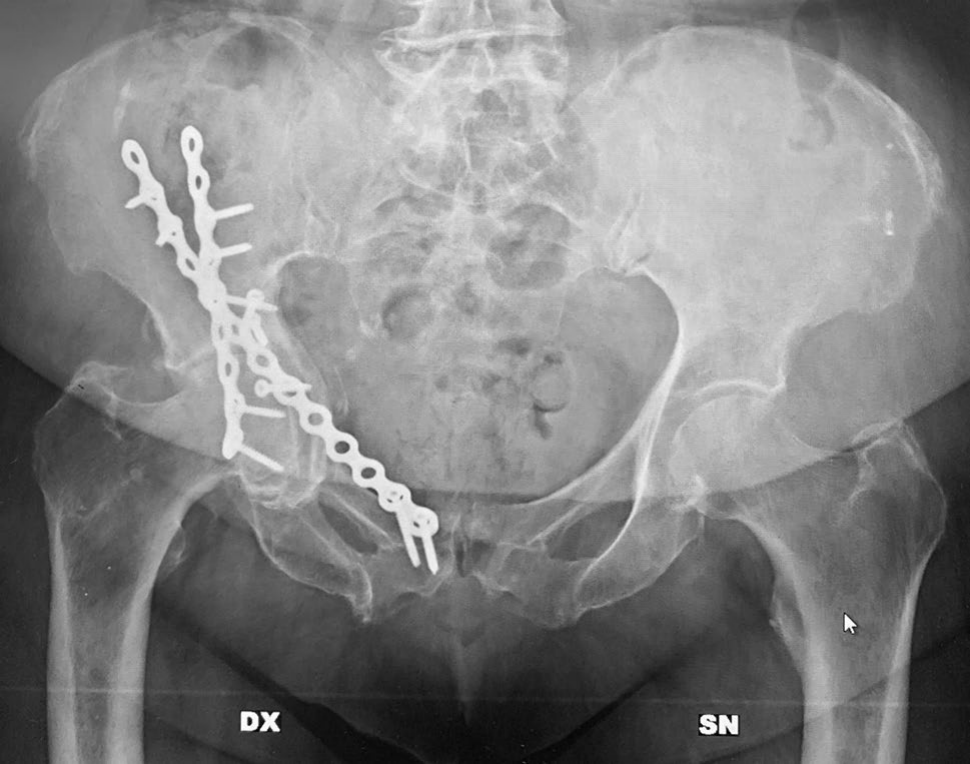
An anteroposterior X-ray in a 78-year-old lady. As opposite of the previous case, note the anatomical reduction of the quadrilateral plate that led to fracture healing without the further development of the osteoarthritis

Considering the poor bone quality, commonly observed in primary osteoporosis as well as in other metabolic bone disease [32], fixation stability is another issue of concern in FFA. Some precautions might be used to overcome this issue. The addition of plate fixation to lag screws improves fixation stability, with locking plates that were specifically developed for the osteoporotic bone [4, 33, 34]. Although the cement augmentation is widely used for improving fixation stability in the osteoporotic bone throughout the body [35, 36], to the best of our knowledge, it has not been used for acetabular fractures. However, nevertheless the type of plate used (conventional or locked), a buttress plate is recommended in case of wall fractures, whereas a neutralization plate for column ones. In posterior column fractures, the use of two buttress plates provides a better fracture stability [37], probably also because the possibility of using a higher screw density in the areas of high bone density. The involvement of quadrilateral plate is a relevant issue considering that its management might be extremely difficult [38, 39]. Figure 2 shows a clinical case representing a failed fixation due to quadrilateral plate reduction loosing. In fact, the quadrilateral plate needs an optimal reduction of the medial fragment protrusion to assure viable outcomes (see Fig. 3). A biomechanical analysis conducted by Culemann et al., analysed different types of fixation of the quadrilateral plate (including conventional, locking and specifically designed plates) showing that conventional plates with three periarticular screws provides the best fixation stability [40]. Anyway, quadrilateral plate non-anatomical reduction could be considered acceptable in elderly patients when the femoral head remains centered within the acetabular roof [41].

##### Percutaneous Fixation

**Fig. 4:**
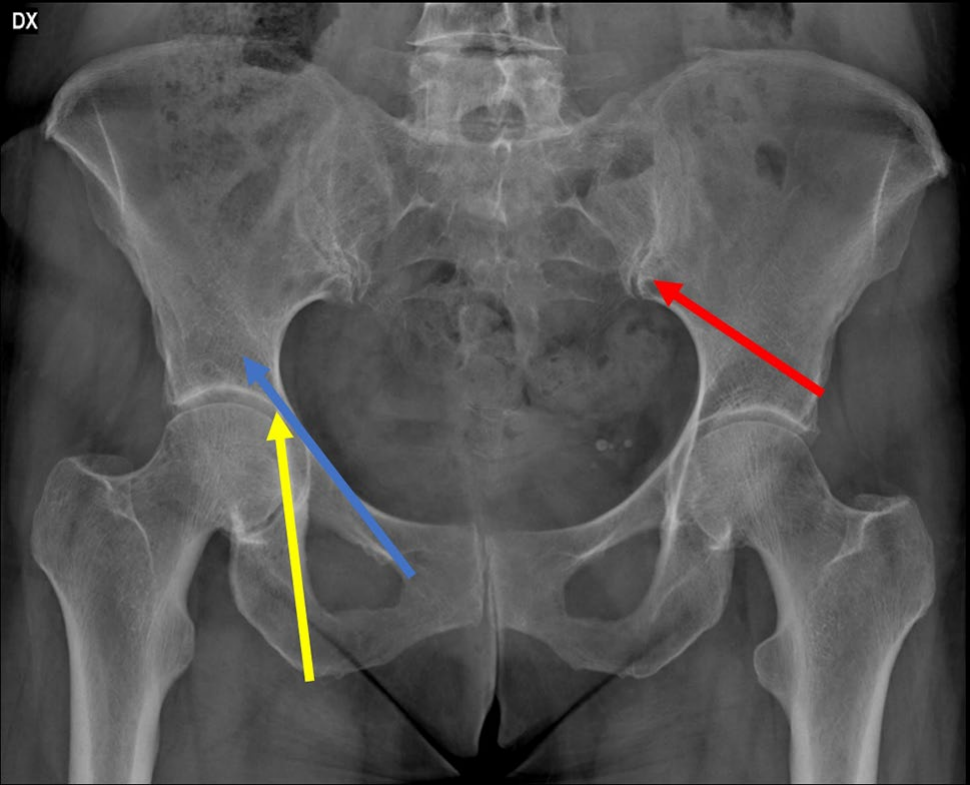
Antero-posterior standard X-ray, showing the three corridors for the percutaneous fixation of the acetabular fractures. In Blue, the iliac-pubic corridor (in retrograde fashion) for the anterior column. In Yellow, the iliac-ischiatic corridor for the fixation for the posterior column. In Red, the dome corridor

Percutaneous fixation of FFA is a challenging technique that could offer some advantages in a subset of patients. In fact, quick recovery, minimal blood loss and a low risk of post-operative infection are achievable, using appropriate small stab incisions to insert modified clamps and pushers for fracture’s reduction and cannulated screw for fixation [42, 43]. Another theoretical advantage of percutaneous fixation is the possibility to perform a subsequent arthroplasty with less technical problems thanks to a limited scar tissue and soft tissue damage. The insertion of the percutaneous screws that fixes the bone is made through 3 osseous “corridors”: the iliac-pubic, the iliac-ischial and the dome zone (see Fig. 4 for further details). Generally, in case of simple column fracture, the stabilization of one corridor is sufficient to fix the fracture, while two to three corridors must be stabilized to fix complex fractures [43]. However, some important issues limit the use of these techniques. In fact, they need a considerable expertise and a relevant confidence with the radiological anatomy of the pelvis, considering the lack of direct visibility of the fracture site. Moreover, an accurate fracture reduction might be difficult to obtain and the screws might be far from the correct and safe positions for the fracture stabilization [12]. Specific contraindications to the percutaneous fixation include posterior wall fractures with hip instability and lack of surgeon expertise with percutaneous pelvic fixation [43].

Table 2 summarizes the available treatment options and their drawbacks in FFA.

**Table 2:** Type of treatment, possible drawbacks, and their solutions

Type of treatment	Drawbacks	Possible solutions
Non-operative	Bed rest complications	Early mobilization; partial weight-bearing as soon as possible
	Secondary fracture displacement	Routine radiograph evaluation; operative treatment
	Secondary osteoarthritis	Total hip arthroplasty
ORIF	Poor bone quality	Neutralization plates OR Locking plates
	Quadrilateral plate displacement	Reduction and plate fixation with 3 periarticular screws OR specific designed plate fixation for quadrilateral plate
	Surgical-related complications in high-risk patients	Accept non-anatomical reduction; Prefer non-extensile surgical approaches
	Secondary osteoarthritis	Total hip arthroplasty
Percutaneous fixation	Technical demanding	Proper knowledge of the radiological anatomy of the pelvis
	Inappropriate screw positioning	Accurate fracture reduction
	Secondary osteoarthritis	Total hip arthroplasty

*ORIF* Open reduction and internal fixation

#### Hip Replacement

Hip osteoarthritis (HOA) is another critical issue that the orthopaedic surgeon has to face up in FFA. In fact, in the elderly, HOA could represent both a late complication and a concomitant disease of the acetabular fracture. Therefore, the use of total hip arthroplasty (THA) is an option that has to be considered, in both the acute setting and fracture sequelae. In Table 3, a summary of the possible pitfalls associated with THA in FFA.

**Table 3:** THA after FFA, possible drawbacks, and their solutions

Type of THA	Drawbacks	Possible solutions
Acute with ORIF	Inadequate cup stability	Column reconstruction + multi-hole revision shell
	Bone loss	Allograft/autograft
	Poor bone quality	Porous metal cups
	Surgical-related complications in high-risk patients	Non-anatomical reduction; proper patient selection
	Poor long-term implant survivorship	Proper patient and implant selection
Delayed after non-operative treatment	THA in non-union or malunion	Accurate evaluation of the preoperative CT scan + appropriate surgical approach + revision cages; plate fixation; bone graft; porous buttress augmentation devices
	Bone loss	Revision cages; bone graft; porous buttress augmentation devices
	Poor femoral bone quality	Cemented stems
Delayed after operative treatment	Infection	Rule out a possible unknown infection preoperatively (i.e.: perform biochemical evaluations)
	Scar tissue and avascularity of the soft tissues	Appropriate surgical approach; proper patient selection
	Bone loss	Revision cages; bone graft; porous buttress augmentation devices
	Hardware retention	Plan to remove hardware coming on the way of THA

*THA* Total hip replacement, *FFA* Fragility Fracture of the Acetabulum, *ORIF* Open reduction and internal fixation

##### Acute THA Associated with Fracture Fixation

The main indications for acute THA associated with fracture fixation are acetabular dome impaction > 40%, concomitant femoral head impaction/neck fracture, acetabular comminution, concomitant HOA and/or multiple associated fractures [44–46]. The goal of fracture fixation in this type of treatment is represented by a rigid stabilization of the fracture without considering the quality of reduction to assure good primary stability to the THA [46]. Typically, this combined procedure could be performed through a Kocher–Lagenbeck approach, but a secondary anterior approach should be done if an anterior fixation is also required.

The acetabular reaming should be made carefully, considering the poor bone quality. The bone defects observable after fracture reduction should be filled with allograft or autograft (i.e., femoral head morselized graft). A multi-hole revision acetabular shell could be useful to achieve additional fixation. The more recently introduced porous metal cups provides sufficient primary stability ensuring good clinical results at mid-term follow-up [47]. The femoral stem implantation is done standardly, using both uncemented and or cemented stems depending on patient’ age, bone quality and surgeon preference [5, 18, 46]. Generally, a bed to chair transfer can be started from the first day after the surgery, while a partial weight-bearing can be allowed from the second with a progressive weight-bearing [29].

However, the indication for this kind of surgery depends also on patient’s general health status. Acute THA has been demonstrated to provide benefit of immediate postoperative weight-bearing, reducing the risk of any thrombotic events, decubitus ulcers and pulmonary complications [48]. However, the procedure might be very challenging and time consuming. Moreover, the 10-year survivorship of this kind if implants is inferior compared to that observed in patients who underwent to THA for primary HOA or avascular necrosis (AVN) [48]. Therefore, a strict patient’s selection is required to assure better outcomes of acute THA in fragility acetabular fractures.

##### Delayed Total Hip Arthroplasty

A delayed THA might be extremely difficult. Therefore, a meticulous pre-operative planning is mandatory. Any bone defect should be analysed using the CT scan [14], and any potential joint infection should be ruled out especially in case of previous ORIF or percutaneous fixation, evaluating at least the serum biomarkers (i.e. erythrocyte sedimentation rate, C-reactive protein, white cell blood count) [49, 50] . The use of revision THA (rTHA) facilities and approaches might be required to face up all the issues related to a delayed THA.

rTHA and THA after ORIF share several issues, including longer operative time, higher blood loss and transfusion rate, presence of scar tissues, avascularity of soft tissues and bone, heterotopic ossification, retained hardware, acetabular deformity and bone loss, and high risk of infection [51]. Considering the above risk factors, the clinical outcomes are less favourable, and a higher revision rate has to be expected following a delayed THA compared to hip replacement for primary HOA [52, 53].

The occurrence of post-traumatic HOA or AVN with the development of a painful hip joint with a decreased quality of life is one of the drawbacks of non-operative treatment of acetabular fractures [52, 53]. Although in this scenario, there are no soft tissue alteration, the technical problems are still relevant, and represented by acetabulum malalignment, severe bone loss and fracture non-union. Therefore, also in case of the occurrence of an HOA after an FFA, conservatively treated rTHA facilities are required. Particularly, cages and bone grafts might be extremely useful [52]. The choice of the surgical approach depends on the surgeon expertise and the type of sequelae. In fact, in case of severe anatomical alterations or instability of the pelvic ring due to fracture non-union (a condition that required the correction of the pelvic ring alignment before performing THA), modified Kocher–Langenbeck or iliofemoral might be preferred [17, 18, 54, 55].

The filling of acetabular bone gaps is one of the most relevant problems observed in these patients, especially in case of central protrusion of the femoral head with the consequent possible excessive medialisation of the implant. Recently, the use of porous tantalum buttress augmentation and tantalum acetabular component has been proposed to decrease the need of cementation and massive bone grafting while increasing the implant stability [56–58]. Another possibility, is to use autologous graft from the patient’s femoral head [8, 59]. The use of impacted morselised allograft and a cemented cup, eventually with a reinforcement ring, could be another viable option to face up the problem of the bone loss [60, 61].

On the femoral side, the use of cemented stems should be preferred because of the predisposition of premature loosening consequently to the long period of inactivity after hip fracture that might lead to a disuse osteoporosis [18].

## Patient’s Outcomes

Confusing outcomes had been reported in FFA, further underlining the difficulties that the orthopaedic surgeon face up in these patients. However, some risk factors for both reoperation need and poor outcomes had been identified (see Table 4) [62, 63].

**Table 4:** Factors associated with worse outcomes

Patient-related	Age Osteoporosis Low activity level Contralateral THA Concomitant HOA
Fracture-related	Roof impaction (gull sign) Quadrilateral plate comminution Posterior wall fragmentation Concomitant femoral head fracture
Surgery-related	Non-anatomical reduction (especially of the quadrilateral plate) Time consuming procedure

*THA* Total hip arthroplasty, *HOA* Hip Osteoarthritis

A systematic review performed by Daurka et al. [9], compared ORIF vs percutaneous, ORIF + THA vs ORIF/Percutaneous and ORIF Vs Conservative. The authors observed several controversies in the achievable outcomes and complications [9]. Particularly, ORIF + THA was the treatment associated with the highest and earliest mortality rate, whereas percutaneous fixation presented a higher mortality rate than ORIF. This surprising observation might be related to the older age of the patients included in that group. These results were further confirmed by the direct comparison through the pooled data between ORIF with ORIF + THA (death occurred at 47.2 months vs 33.3 months respectively) and ORIF vs percutaneous (death occurred at 47.2 months vs 121.8 months respectively) (see Table 1 for further details). Of note, a high conversion rate to THA was reported by the authors regardless of the fixation technique used (22% ORIF vs 25% percutaneous). Despite performing THA in patients with acetabular fractures is a complex procedure, it provides significant improvement in pain and function. In fact, Stibolt et al. [52] in a review including 448 patients with a median age of 51.5, observed that delayed THA was associated to an increase of the mean Harris Hip Score from 41.5 to 87.6 at a mean of 82 months of follow-up.

However, the complication rate is generally high (i.e. infections, sciatic nerve palsy, dislocation, heterotopic ossification) and THA in acetabular fracture was associated to an inferior 10-year survival compared to those with primary OA or AVN [64].

Finally, a recent study conducted by Glogovac et al. observed that performing the surgery within the first 48 h after the injury were not associated with mortality rates benefits in elderlies with acetabular fractures, in contrast to that reported for proximal femur fractures [65]. The authors concluded that the time to surgery in acetabular fragility fractures should be determined on individual bases [65].

## FFA and Osteoporosis Management

Very few studies evaluated the efficacy of anti-osteoporotic drugs in FFA, however, as well as the other fragility fractures an appropriate management must also consider the underlying osteoporosis and sarcopenia to improve outcomes and prevent subsequent fractures [66–68]. According to the clinical guidelines of the Italian Society for Orthopaedics and Traumatology to appropriately manage a patient with an osteoporotic fracture, a comprehensive evaluation of the clinical risk factors, and an evaluation of the bone metabolism through both biochemical testing and DXA scan might be performed [66]. After excluding secondary osteoporosis, the treatment would be based mainly on vitamin D and/or calcium supplementations and both antiresorptive and anabolic anti-osteoporotic drugs [66]. Bisphosphonates and denosumab are well established and safe antiresorptive drugs with proved efficacy in preventing fragility fractures [66, 67, 69–71]. However, some concerns had been raised around their long-term safety, considering that their use had been associated with osteonecrosis of the jaw and atypical femoral fractures [72–74]. Therefore, an accurate follow-up of patients under anti-resorptive drugs is mandatory to timely correct the therapy, if required [66, 75]. Anabolic drugs, like teriparatide or romosozumab, are generally used in non-responsive patients to antiresorptive therapy (i.e.: occurrence of a new fragility fractures in patients under anti-resorptive drugs) [76–78]. Moreover, the availability of anabolic drugs paved the way to the implementation of sequential therapies strategies (i.e. antiresorptive therapy first followed by an anabolic drug; anabolic therapy first followed by an antiresorptive drug, co-administration of both antiresorptive and anabolic agents) [78, 79]. Among them, the administration of an anabolic drug followed by an antiresorptive one seems to be associated with the best outcomes in terms of fragility fracture prevention [78].

Interestingly, the use of anti-osteoporotic drugs (both vitamin D supplementation and bisphosphonates) in patients with FFA was associated to a reduction in the time to fracture healing [68]. According to the authors, the osteoporosis management as a part of a standardized protocol for the management of FFA focused also on the optimisation of medical comorbidities, early ambulation, and early hospital discharge to an appropriate facility, was associated to an improvement in functional outcomes [68].

**Fig. 5:**
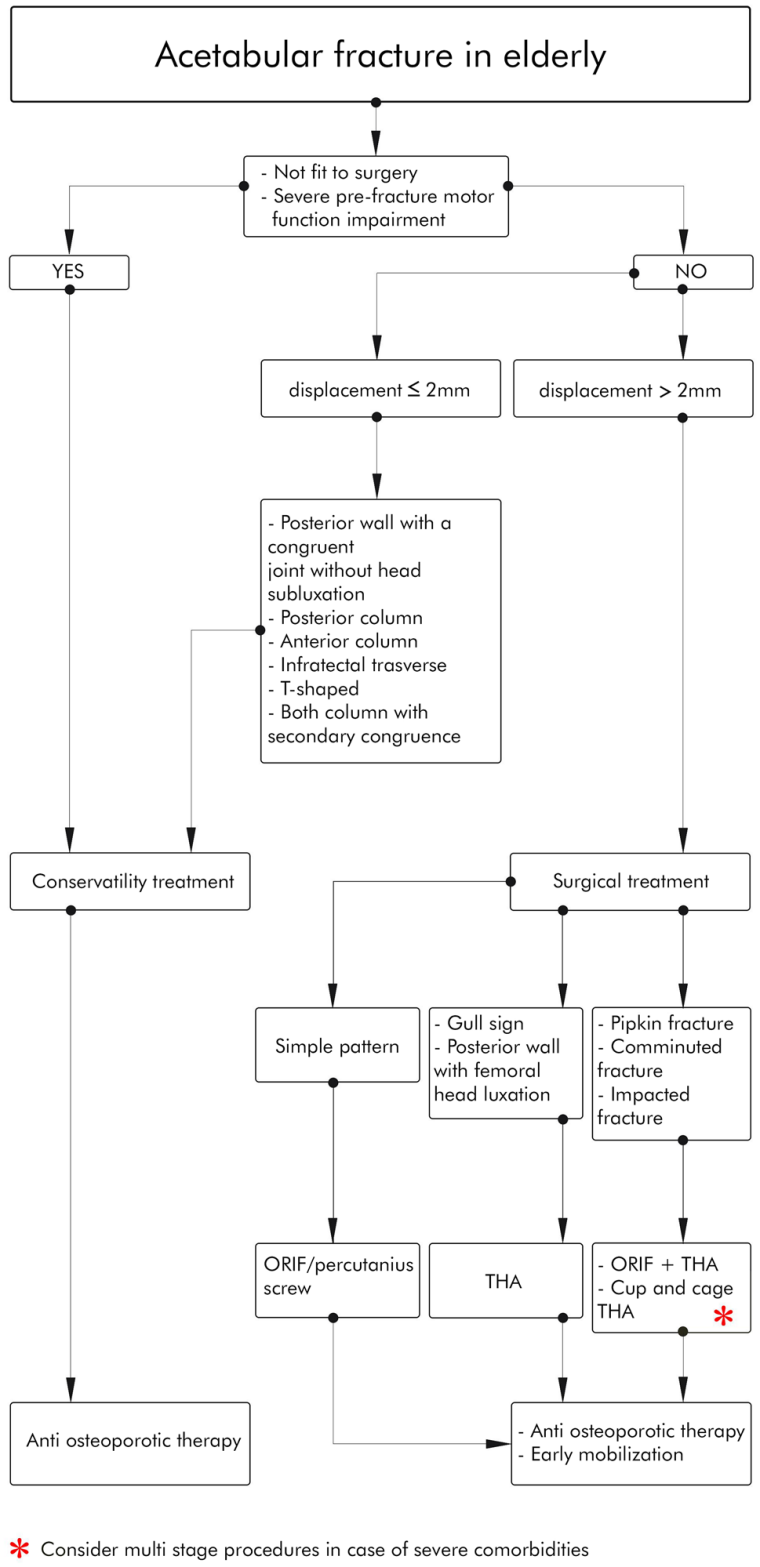
Treatment algorithm for a practical approach to FFA

In Fig. 5, we proposed an algorithm for a practical approach to FFA.

## Conclusion

Acetabular fractures in the elderly are constantly increasing during last years and requires a comprehensive patient’s evaluation and advanced surgical skills. The treatment choice is exceptionally difficult, considering the controverted outcomes and high rate of complications reported in the available literature. ORIF could provide good clinical outcomes when a congruent, anatomic reduction is obtained. However, an intermediate mortality risk had been associated with ORIF and a large part of patients would require a subsequent THA.

A primary THA with a concomitant ORIF might be preferable for patients with simple fractures and severe comorbidities for whom a second surgery could be not affordable. However, this kind of approach was associated to the earliest mortality.

Although theoretically less risky for the patient, a special attention in the preoperative planning is mandatory in case of delayed THA, because of the high complexity of this procedure in comparison with an implantation after a primary OA.

Finally, in our opinion, in the elderly, the golden rule of “*primum non nocere*” is more relevant than gaining perfect reductions, which may lead to more viable long-term outcomes, or performing complex reconstruction to achieve quicker recovery. Therefore, the surgeon must consider both fracture pattern and patients’ health status (including comorbidities and pre-fracture walking abilities) for the treatment choice, eventually preferring staged procedures. As well as other fragility fractures, a multidisciplinary treatment based on a standardized protocol to ensure a holistic approach to FFA, with the final purpose of improve patients’ outcomes, may be advisable.
